# The Impact and Treatment of COVID-19 in Hemodialysis Patients

**DOI:** 10.3390/jcm12030838

**Published:** 2023-01-20

**Authors:** Daisuke Katagiri, Kan Kikuchi

**Affiliations:** 1 Department of Nephrology, National Center for Global Health and Medicine, Tokyo 162-8655, Japan; 2 Division of Nephrology, Shimoochiai Clinic, Tokyo 161-0033, Japan

**Keywords:** COVID-19, hemodialysis, vaccination

## Abstract

Background: Patients with coronavirus disease 2019 (COVID-19) undergoing maintenance hemodialysis have a poor prognosis and limited treatment options. Methods: This paper outlines the impact of COVID-19, its treatment, and the efficacy of vaccines in Japanese patients undergoing hemodialysis with a review of the literature. Results: Patients undergoing dialysis in dialysis facilities are at greater risk of exposure to severe acute respiratory syndrome coronavirus 2 than the general population due to limited isolation capabilities. Therefore, vaccines are expected to be effective for patients undergoing dialysis. In addition, effective use of available medications is important because treatment options are limited. Conclusions: Efforts should be made to prevent the spread of the infection to high-risk patients undergoing dialysis while ensuring the effective use of vaccines.

## 1. Introduction

In December 2019, an outbreak of unknown viral pneumonia was reported among patients in Wuhan, Hubei Province, People’s Republic of China. Over a short period, infection by a novel severe acute respiratory syndrome coronavirus 2 (SARS-CoV-2) spread worldwide. SARS-CoV-2 has been identified as an animal-derived coronavirus and is the same pathogen responsible for severe acute respiratory syndrome (SARS) and Middle East respiratory syndrome (MERS). On 11 March 2020, the World Health Organization (WHO) declared a pandemic status, and SARS-CoV-2 was linked to a severe acute respiratory condition. SARS-CoV-2 has been reported to be stable on environmental surfaces for approximately 3 days. Therefore, preventing infection among staff is crucial in medical institutions.

Initial coronavirus disease 2019 (COVID-19) symptoms are similar to those of influenza, including fever, cough, malaise, and dyspnea. The median hospitalization time was 7 days. Diarrhea and taste and smell disorders may occur; however, they are not inevitable. Data from the COVID-19 Registry Japan (COVIREGI-JP), which is a Japanese registry of patients with COVID-19, showed that 60% of hospitalized patients did not require oxygen administration, whereas 30% required oxygen administration, 9% required ventilation or extracorporeal membrane oxygenation (ECMO), and 7.5% died [1]. Therefore, predicting which patients will become critically ill is important for using limited medical resources [2].

In severe cases, COVID-19 causes respiratory tract infection symptoms, such as acute respiratory distress syndrome and cytokine release syndrome (CRS)-like symptoms because of excessive inflammation. Endothelial cell damage and disruption of the immunomodulatory system lead to multiple organs failure. During the COVID-19 pandemic, the number of dialysis cases in the hospital was also reported to have increased greatly, partly due to the involvement of acute kidney injury [3]. Therefore, in addition to antiviral drugs, various therapies have been investigated to suppress excess cytokines, such as steroids, neutralizing antibody therapy, and some blood purification therapies [4,5]. However, patients undergoing dialysis are prone to severe disease, and their treatment options are limited because of renal dysfunction. This manuscript outlines the current impact of COVID-19 and its treatment in Japanese patients undergoing dialysis.

## 2. Number and Severity of Patients with COVID-19 Undergoing Dialysis in Japan

**Figure 1:**
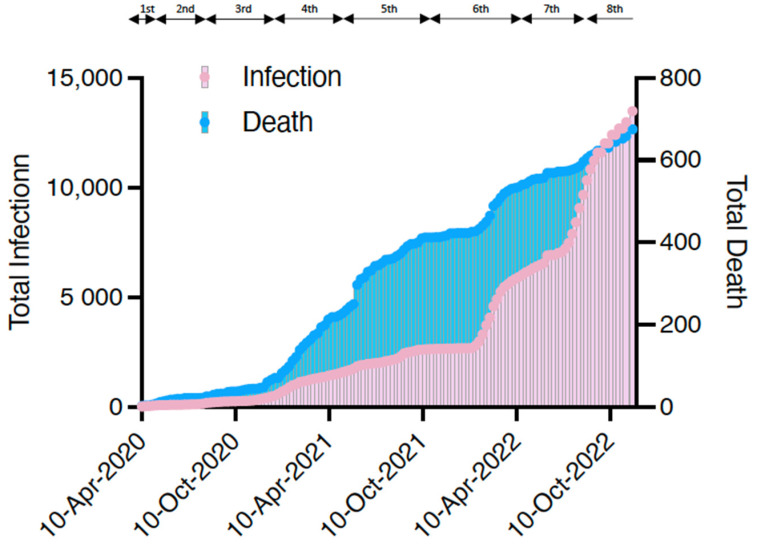
The total number of infected patients undergoing dialysis in Japan and the number of deaths. The periods of the first to eighth waves (the 8th wave is ongoing) are also shown. Data were taken from the website of the Japanese Association of Dialysis Physicians (http://www.touseki-ikai.or.jp/, accessed on 7 December 2022) and plotted.

**Figure 2:**
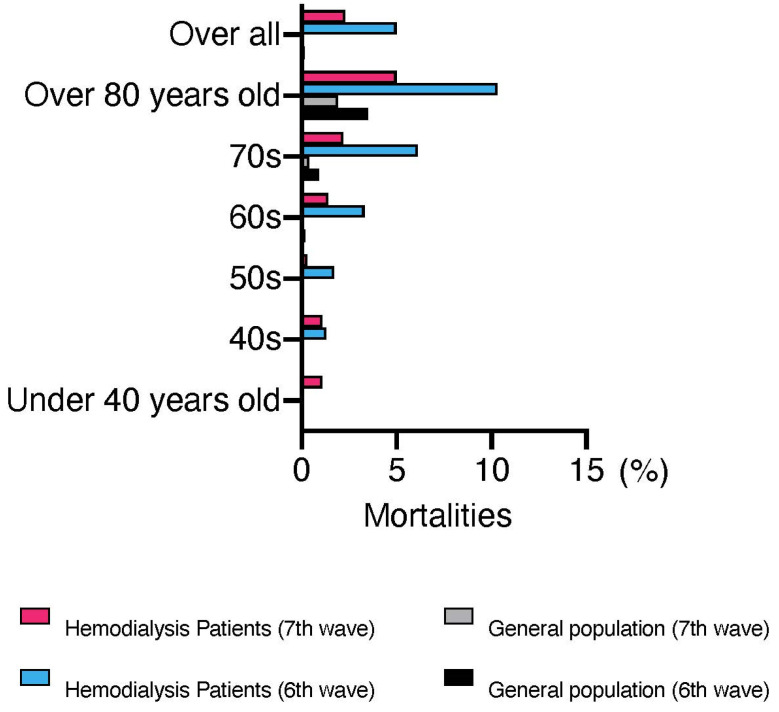
Mortalities in the general population and patients undergoing dialysis after the sixth wave. This figure was drawn from the 9 November 2022 report of the Advisory Board for New Coronavirus Infections (https://www.mhlw.go.jp/content/10900000/001010896.pdf, accessed on 7 December 2022) and the “Report on COVID-19 Infection Cases at Dialysis Facilities” jointly published by the Japanese Association of Dialysis Physicians, the Japanese Society for Dialysis Therapy, and the Japanese Society of Nephrology. This figure is accurate as of 7 December 2022.

The first case of COVID-19 in a patient undergoing dialysis was reported in Japan on 1 March 2020 [6]. The Japanese Association of Dialysis Physicians, the Japanese Society for Dialysis Therapy, and the Japanese Society of Nephrology established the Joint Committee on Countermeasures against SARS-CoV-2 infection in Dialysis Patients to monitor the infection status of patients undergoing dialysis in Japan. As shown in Figure 1, the number of infected patients on dialysis continues to increase in Japan. Although the number of deaths appears to have decreased compared with the past, partly due to the spread of vaccines, continued attention should be given in the future. As of November 2022, the total number of infected patients was 12,978, and the infection rate was 3.8% of the total number of patients on maintenance dialysis in Japan (approximately 340,000). Overall, 658 confirmed deaths have been recorded due to COVID-19 among patients undergoing dialysis, with a mortality rate of 5.1% higher than that in the general population (0.2%). Figure 2 shows that even after the virus mutated to Omicron, the mortality rate among patients undergoing dialysis remained higher than that of the general population, particularly among those aged <60 years.

## 3. Efficacy of COVID-19 Vaccination in Patients with End-Stage Renal Disease

During the first to fourth waves, vaccines had not yet been developed and disseminated in Japan; however, they became widespread during the fifth wave. The weakening of the virus may have played a role in the significant decrease in severe cases and deaths among patients undergoing dialysis. However, these patients remain at high risk compared with the general population, as shown above.

Several studies have analyzed the efficacy and safety of the COVID-19 vaccine among patients undergoing hemodialysis [7,8]. A study of 148 and 20 patients undergoing hemodialysis and peritoneal dialysis, respectively, reported similar efficacy of COVID-19 mRNA vaccination [9]. Although caution must be exercised during interpretation due to the heterogeneous study design, in most studies, humoral responses were lower than that in the control group. In contrast, seroconversion rates and the number of patients in whom S-protein reactive T-cell immunity was detected, were very high [10]. On 15 October 2021, the American Society of Nephrology released a statement on the need for vaccines for patients undergoing dialysis [11]. The report emphasizes the importance of vaccination in patients with end-stage renal disease (ESRD) to reduce the increased risk of complications and death secondary to COVID-19 infection. In addition, patients with end-stage kidney disease and kidney transplantation have a reduced antibody response to the COVID-19 vaccine; however, antibody production has been shown to increase with the third and fourth doses [12]. Multivariate logistic regression analysis was used to examine post-infection oxygen demand in patients with post-vaccinated infection and breakthrough infection in Japan [13]. The odds ratio (OR) 0.197 (95% confidence interval [CI]: 0.120–0.322), *p* < 0.001, showed that patients with breakthrough infection had lower oxygen demand. The prognosis of breakthrough-infected patients was also better than that of unvaccinated patients.

## 4. Infection Control and Hospitalization of Patients Undergoing Dialysis in Japan

Patients receiving dialysis at the center were at greater risk of exposure to SARS-CoV-2 than the general population because of their limited isolation capabilities [14]. They were initially required by national policy to be hospitalized because of the high mortality rate associated with COVID-19 [6]. A report from Canada showed that the rate of hospitalization, 30-day mortality, and overall mortality were all significantly lower in patients receiving home dialysis, including patients undergoing peritoneal dialysis, than in those undergoing outpatient hemodialysis [15]. However, an analysis by Kikuchi et al. based on Japanese registries comparing patients receiving peritoneal dialysis and hemodialysis in terms of overall survival and length of hospitalization showed no significant difference between the two groups [16]. Therefore, caution should be exercised because overly strict infection precautions increase the burden on staff, increase healthcare costs, and make compliance more challenging. Infection control in patients undergoing dialysis has traditionally been well-established in Japan, and the 5th edition of the guidelines was issued in 2020 [17]. The following is a list of infection control measures that are in place at dialysis facilities in Japan regularly:

(1) Personal protective equipment (PPE) is recommended for the medical staff in the dialysis unit.

Before performing procedures such as puncture, hemostasis, catheter access and management, and wound care, hand hygiene should be performed by washing hands with soap and running water or using a quick-drying hand sanitizer, and unused disposable gloves should be worn. In addition, wear a disposable nonpermeable gown or plastic apron, surgical mask, goggles, or face shield when performing procedures such as puncture, hemostasis, catheter access and management, and wound care.

(2) Environmental hygiene in the dialysis unit.

Linens (sheets, pillowcases, and blanket covers) should be changed for each patient. The exterior of the dialysis machine, bed rails, and over tables should be cleaned at the end of each dialysis session. Stethoscopes, thermometers, and blood pressure cuffs should be cleaned after each use. Instruments in the dialysis room should be cleaned and disinfected with either 0.05–0.1% sodium hypochlorite, potassium hydrogen peroxymonosulfate, or alcohol-based disinfectants. Forceps and trays, among others, should be disinfected with hot water (80 °C for 10 min) or thoroughly pre-cleaned with a cleaning agent before each use, immersed in 0.1% sodium hypochlorite for 30 min, and then thoroughly rinsed with water. The above infection control measures are recommended in normal times, and the use of PPE and environmental sanitation are also preventive measures against contact and droplet infection of COVID-19.

A survey of dialysis facilities in Japan [18] revealed that several infection prevention measures were implemented during the COVID-19 pandemic, including health checks of staff and patients, wearing of masks before and after hemodialysis, and disinfection of frequently contacted areas. The implementation rate of these measures was significantly improved compared with that of the pre-pandemic rate, reaching ˃90%. However, because of the high risk of infectious disease transmission in the hospital setting during a pandemic, alternative end-stage renal failure management methods may need to be considered, such as a temporary switch to peritoneal dialysis or the implementation of a home dialysis program [19].

As noted above, the Japanese government recommended that patients who tested positive be hospitalized because of the high mortality rate associated with COVID-19, particularly patients receiving maintenance dialysis and those with a definite need for regular dialysis. However, after experiencing a delta surge, a strategic shift to outpatient care for mildly ill or asymptomatic patients and increased emergency preparedness was necessary. In response to the rise in the Omicron variant, the Tokyo Metropolitan Government opened a temporary medical facility with a dialysis center in January 2022, providing more beds and access to hemodialysis [20]. The hospital ran a smooth ward operation and reduced the number of complications with new patients with positive COVID-19 test results that required treatment and could not be hospitalized.

## 5. Current Treatment of COVID-19 in Patients Undergoing Dialysis

As of November 2022, the antivirals approved in Japan for treating COVID-19 include remdesivir, molnupiravir, nirmatrelvir/ritonavir, and the recently approved ensitrelvir (Table 1).

Remdesivir is recommended for mild-to-moderate disease within 7 days of onset. Japanese patients with COVID-19 undergoing hemodialysis enrolled by 19 June 2020, with (*N* = 98) and without (*N* = 294) remdesivir, were studied using propensity matching [16]. Patients receiving remdesivir had a significantly better prognosis than those not receiving it. In addition, the remdesivir-treated group had a shorter hospital stay. In a retrospective study of 486 patients (407 on hemodialysis and 79 on peritoneal dialysis) in the United States, 112 (23%) received remdesivir [21]. The estimated 30-day mortality rate was 0.74 (95% confidence interval, 0.52–1.05) in the remdesivir-treated group compared with the non-treated group. These results suggest that remdesivir is an effective treatment option for patients undergoing maintenance hemodialysis.

Molnupiravir was the first oral antiviral drug approved in Japan to treat COVID-19 [22]. It does not require dosage adjustment according to renal function or volume adjustment in patients undergoing hemodialysis, making it easy to use in outpatient settings [23]. However, the disadvantage is that the capsule formulation is large and challenging to take internally. Nirmatrelvir/ritonavir is another oral antiviral drug approved in Japan [24]. As ritonavir inhibits drug metabolism in CYP3A to maintain drug blood levels, it increases the blood levels of drugs metabolized by CYP3A. Calcium channel blockers and statins are typical examples, but many other drugs, such as tranquilizers, are also affected. Dose adjustment is required in patients with moderately impaired renal function, and administration is not recommended for patients with severe renal dysfunction, including those on maintenance dialysis. Clinical trials have not been conducted on ensitrelvir in patients with renal dysfunction, and its efficacy in those undergoing hemodialysis requires further study.

Omicron strains have been classified into five strains (BA.1, BA.2, BA.3, BA.4, and BA.5). The BA.2 strain has been the primary epidemic strain; however, since July 2022, the BA.2 strain has been rapidly replaced by the BA.5 strain in many countries, including Japan. The inhibitory effects of different antibodies and antiviral drugs on Omicron strains isolated from clinical specimens are being investigated [25]. The neutralizing activity of sotrovimab and casirivimab–imdevimab [26] was significantly lower against all strains after BA.2 than the effect against the conventional stress (from Wuhan). The efficacy of tixagevimab and cilgavimab was similarly reduced. In contrast, bebtelovimab showed a high neutralizing activity against BA.2.12.1, BA.4, and BA.5 strains. Furthermore, the efficacy of the three antiviral drugs (remdesivir, molnupiravir, and nirmatrelvir) was subsequently analyzed, and they were found to effectively inhibit the growth of BA.2.12.1, BA.4, and BA.5 strains.

## 6. Conclusions

This paper outlines the impact and treatment of COVID-19 on patients undergoing hemodialysis, which has not yet reached a global consensus. Therefore, it is important to continue to elucidate the pathogenesis of severe disease in patients with hemodialysis, leading to expanded vaccination and the establishment of more effective treatment strategies.

## Figures and Tables

**Table 1:** Current COVID-19 treatment for patients undergoing dialysis.

Type	Antiviral Drug				Immunity Suppressants/Regulators			Neutralizing Antibody	
Name	Remdesivir	Molnupiravir	Nirmatrelvir/ Ritonavir	Ensitrelvir	Dexamethasone	Baricitinib	Tocilizumab	Sotrovimab	Casirivimab–imdevimab
Severity to be administered	Mild, Moderate Severe	Mild, Moderate	Mild, Moderate	Mild, Moderate	Moderate, Severe	Moderate, Severe	Moderate, Severe	Mild	Mild
Response to Omicron	Yes	Yes	Yes	Yes	Yes	Yes	Yes	No	No
Route of administration	Intravenous	Oral	Oral	Oral	Intravenous, oral	Oral	Intravenous	Intravenous	Intravenous
Length of treatment	3–10 days	5 days	5 days	5 days	10 days	14 days	Single dose	Single dose	Single dose
Dosage in dialysis patients	100 mg 4 h before dialysis initiation. Approximately 6 doses	No adjustment required	No administration to dialysis patients	No clinical trials have been conducted in patients with renal dysfunction	No adjustment required	No administration to dialysis patients	No adjustment required	No adjustment required	No adjustment required

## Data Availability

Not applicable.
